# β-Lactamases: Sequence, Structure, Function, and Inhibition

**DOI:** 10.3390/biom11070986

**Published:** 2021-07-05

**Authors:** Peter Oelschlaeger

**Affiliations:** Department of Pharmaceutical Sciences, College of Pharmacy, Western University of Health Sciences, Pomona, CA 91766, USA; poelschlaeger@westernu.edu

β-Lactams were the first class of antibiotics to be discovered and the second to be introduced into the clinic in the 1940s [1]. They inhibit bacterial transpeptidases (also known as penicillin-binding proteins or PBPs) involved in peptidoglycan synthesis, thus inhibiting growth, and leading to lysis of bacteria. The action of β-lactamases, enzymes that hydrolyze and inactivate β-lactams, has been known since 1940 [2], and phylogenetic studies suggest that some of these enzymes have their origin more than 2 billion years ago [3]. Over the past eight decades, β-lactamases have come into focus as factors of antibiotic resistance. For any β-lactam currently available in the clinic, there is a β-lactamase that can inactivate it. As a result, there has been a lot of interest in learning more about how these enzymes function, evolve, are transferred, and can be inhibited.

Thirteen contributions were published in this Special Issue, including three reviews and ten original research articles or communications spanning all four classes of β-lactamases.

In their review that was selected Editor’s Choice, Ramirez, Bonomo & Tolmasky [4] give a detailed account of carbapenemases found in *Acinetobacter baumannii*. *Acinetobacter* spp. are associated with hospital- and community-acquired infections, mostly in patients with preexisting comorbidities. They have an extraordinary genetic plasticity, which has contributed to their ability to resist a variety of disinfectants and antibiotics, as well as to survive in various environments, including on inert surfaces and medical devices through the formation of biofilms. In particular, the occurrence of carbapenemase-resistant *A. baumannii* (CRAB) is described as a quantum leap in the difficulty to treat these infections. Apart from efflux pumps, a decrease in number or function of porins, and changes in type or expression of PBPs, carbapenemases present the most frequently observed carbapenem resistance mechanism. These enzymes belong to classes A (KPC), B (NDM, VIM, and IMP), and D (OXA) and have contributed to extensively drug-resistant (XDR, resistance to at least three classes of antimicrobials plus carbapenems) and pan-drug-resistant (PDR = XDR plus resistance to polymyxins) strains.

Egorov et al. [5] review the role of the Ω-loop in TEM β-lactamases. These class A enzymes are probably the best-studied β-lactamase family with the description of TEM-1 dating back to the 1960s [6]. Like classes C and D, they employ an active-site serine for their catalytic mechanism. They originally evolved from the transpeptidase (PBP) target of β-lactam antibiotics through the formation of the Ω-loop. Residues E166 and N170 in the Ω-loop facilitate the deacylation step, which is more efficient in serine β-lactamases than in PBPs, resulting in the inactivation of rather than inhibition by the β-lactam. The authors describe the Ω-loop as featuring low mutability, stable topology, and structural flexibility, rendering it a potential target for allosteric inhibitors.

Palacios et al. [7] review several inhibitors of class B enzymes or metallo-β-lactamases (MBLs) inspired by β-lactam species along the catalytic mechanism. Rather than an active-site serine, these enzymes employ a hydroxide that is activated by coordination to the Zn(II) ion(s) as the nucleophile initiating β-lactam hydrolysis. The authors start off their review by highlighting structural similarities and differences between the different MBL subclasses (B1, B2, and B3), comparing their reaction mechanism to that of serine β-lactamases, and then report on various inhibitors that mimic substrates, transition states, intermediates, and products, many of which are thiol- and boron-based inhibitors. Covalent inhibitors, such as ebselen and Zn(II)-displacing agents are discussed as well.

Saliu et al. [8] describe the impact of nutrition-related stress factors on the transfer efficiency of extended-spectrum β-lactamase (ESBL) genes from *E. coli* donor cells to *Salmonella* Typhimurium recipients. Historically, ESBLs are TEM and SHV and, more recently, CTX-M (all class A) variants that have acquired mutations enabling them to hydrolyze extended spectrum β-lactams, specifically oxyimino-cephalosporins and the oxyimino-monobactam aztreonam. As stress factors, the authors chose pH, osmolality, copper, zinc, different acids, and subtherapeutic levels of different antibiotics. They found that increased concentrations of propionic acid, copper, zinc, nitrofurantoin, and increased osmolality reduced conjugation frequency, challenging the hypothesis that stress factors enhance conjugation.

In a virtual screening study aiming at identifying inhibitors of the class A β-lactamase GES-5, Klein et al. [9] used a hierarchical method including docking compounds from a library of commercially available compounds to a crystal structure of GES-5. Forty-four compounds were selected for experimental testing against GES-5 and/or KPC-2 and three showed submillimolar inhibition of both GES-5 and KPC-2. These compounds are from novel chemotypes and can be further developed to increase binding affinity.

There are three medicinal chemistry studies with the goal of finding MBL inhibitors in this issue. Gavara et al. [10] report on a series of 1,2,4-triazole-3-thione compounds, which were tested against the clinically relevant IMP-, VIM-, and NDM-type enzymes. One hydrazine compound, less prone to hydrolysis than hydrazones, showed good activity against VIM-2 and was able to significantly increase the inhibition zone diameter of *E. coli* expressing VIM-2 around a cefoxitin disk in a disk diffusion assay. Its inhibition and binding mode were further characterized through isothermal titration calorimetry (ITC) and a docking study. The latter showed the triazole-thione moiety coordinating the Zn(II) ions.

Xiang et al. [11] also studied inhibition of VIM-2 by similar compounds, 2-triazolylthioacetamides. Several compounds were able to decrease the MIC of cefazolin 4- to 8-fold against VIM-2-expressing *E. coli*. ITC experiments suggested that there were entropic and enthalpic contributions to inhibitor binding. A crystal structure of VIM-2 in complex with one compound at 1.78 Å resolution revealed that the triazole as well as a carboxyl substituent on a phenyl ring attached to the triazole ring coordinated Zn(II) ions.

In a communication by Ge et al. [12] from the same laboratory, the authors explore diothiocarbamates as MBL inhibitors. Some of the molecules from this novel scaffold inhibited MBLs from all three subclasses in the micromolar range and individual enzymes in the submicromolar range. Several molecules were also able to significantly decrease MICs of cefazolin and imipenem against *E. coli* cells expressing the NDM-1, ImiS or L1 enzymes. ITC experiments and docking studies were employed to elucidate the mode of inhibition and binding, respectively. Cytotoxicity assays revealed low toxicity of the compounds. Given the dearth of MBL inhibitors, these three studies are encouraging.

Cheng et al. [13] report on MBLinhibitors.com, a carefully curated database currently consisting of more than 900 MBL inhibitors, which can be searched by chemical name, formula, functional groups, or the MBL that they inhibit. Apart from giving a comprehensive overview of the various inhibitors, the idea is to foster collaboration between researchers searching for MBL inhibitors to improve the likelihood of discovering a clinically useful MBL inhibitor.

An avenue parallel to finding MBL inhibitors is to better understand how MBLs evolve to inactivate β-lactam antibiotics or evade the action of inhibitors. Zhang et al. [14] investigate the role of two mutations (S115T and S119G) in IMP enzymes that always occur in combination. Based on microbiological, expression level, biophysical, and biochemical kinetic analysis of four enzymes carrying none, one of the two, or both mutations in the IMP-1 sequence background, they conclude that the two mutations work together in a concerted fashion; S119G by increasing activity against new-generation cephalosporins and carbapenems, and S115T by restoring thermal stability and high expression level, which were compromised by the introduction of S119G.

Lang et al. [15] report on crystallographic and kinetic experiments with bicyclic boronates as potential inhibitors of class C enzymes, in particular, AmpC from *E. coli*. One such compound, taniborbactam in combination with cefepime, is already in phase 3 clinical trials. Kinetic studies showed that AmpC was inhibited by such compounds in the low micromolar range, and ceftazidime MICs with *E. coli* expressing AmpC were significantly decreased (64- to 512-fold) in the presence of 5 µg/mL of the compounds. A crystal structure revealed that the bicyclic, but not the tricyclic, form of taniborbactam bound to the active site of AmpC as a high-energy tetrahedral intermediate. This study is significant, because it provides evidence of the utility of bicyclic boronates as class C enzyme inhibitors after they have already shown to be potent inhibitors of class A, B, and D enzymes.

More evidence for the utility of boronates as inhibitors of class C enzymes is provided by Lefurgy et al. [16]. They report on four compounds designed as boronic acid transition-state analogs (BATSIs) that were tested against the extended spectrum class C cephalosporinase FOX-4 (named for its substrate preference for the cephamycin cefoxitin). The compounds, especially SM23, which resembles cephalothin, exhibit very low (nanomolar) IC_50_ values against FOX-4 (this study) as well as against *E. coli* AmpC and *P. aeruginosa*-derived cephalosporinase (PDC)-3 (previous studies). SM23, as well as LP-06 with a side chain identical to the ceftazidime R1 side chain, significantly (8- to 128-fold) decreased ceftazidime MICs against *E. coli* expressing FOX-4. Similar to the report by Lang et al., SM23 was also covalently bound to S64 as a tetrahedral intermediate in a crystal structure.

A contribution by Frère et al. [17] discusses the interaction between avibactam and different OXA enzyme variants (class D). Avibactam belongs to the recently introduced diazobicyclooctanones and is currently approved with the extended-spectrum cephalosporin ceftazidime for complicated intra-abdominal infections and complicated urinary tract infections. Some OXA variants have carbapenemase activity but typically low activity against extended-spectrum cephalosporins, such as ceftazidime. Interestingly, OXA-163 has a four-amino acid deletion relative to the carbapenemase OXA-48, which improves its activity against ceftazidime while decreasing its carbapenemase activity. OXA-427 is another, distantly related, variant that has both carbapenemase and ESBL activity. The activity of avibactam against different OXA enzymes varies. Therefore, the authors investigated ceftazidime hydrolysis by and avibactam inactivation of OXA-163 and OXA-427. They conclude that these two enzymes are inhibited quite effectively by avibactam, which is desirable given their ability to hydrolyze the partner drug ceftazidime.

This special issue covers a broad range of methods applied to the study of all four classes of β-lactamases. It is encouraging that there is an advancement of our knowledge base about how these important enzymes are transferred, their function, and how we can inhibit them. At the same time, it is abundantly clear that the incredible diversity of these enzymes with regards to sequence, structure, and catalytic mechanism (which is continuously augmented by their evolution) make the prospects of developing a β-lactam antibiotic that is stable to all these enzymes or a pan-β-lactamase inhibitor an elusive goal, and research in this field will continue to be required.
